# 4366 Aligning community-engaged research competencies with online training resources across the Clinical and Translational Science Award Consortium

**DOI:** 10.1017/cts.2020.253

**Published:** 2020-07-29

**Authors:** Rebecca Jane Piasecki, Rebecca J Piasecki, Lisa D Quarles, Mona N Bahouth, Anwesha Nandi, Alicia Bilheimer, Lori Carter-Edwards, Cheryl R Dennison-Himmelfarb

**Affiliations:** 1 Johns Hopkins University School of Medicine; 2 Johns Hopkins University School of Nursing; 3 North Carolina Translational and Clinical Sciences Institute

## Abstract

OBJECTIVES/GOALS: The extent that Clinical and Translational Science Award (CTSA) programs offer resources accessible online for training in community-engaged research (CEnR) core competencies is unknown. This study cataloged CEnR resources accessible online from CTSAs and mapped resources to CEnR core competencies. METHODS/STUDY POPULATION: Eight domains of CEnR core competencies were identified: knowledge/perceptions of CEnR; personal traits necessary for CEnR; knowledge of/relationships with communities; training for performing CEnR; CEnR methods; program evaluation; resource sharing and communication; and dissemination and advocacy. A systematic review of CEnR resources accessible online from CTSAs was conducted between July 2018 and May 2019. Resource content was independently reviewed by two reviewers and scored for inclusion of each domain of CEnR core competencies. Domain scores across all resources and inter-rater reliability in scoring domains were assessed using descriptive statistics and Cohen’s kappa coefficients. RESULTS/ANTICIPATED RESULTS: Overall, 214 resources available from 24 CTSAs were eligible for full review. Scoring discrepancies for at least one domain within a resource initially occurred in 51% of resources. “CEnR methods” (50.5%; 108 of 214) and “Knowledge of/relationships with the community” (40.2%; 86 of 214) were most frequently addressed and “Program evaluation” (12.1%; 26 of 214) and “Dissemination and advocacy” (11.2%; 24 of 214) were least frequently addressed domains. Additionally, challenges were noted in navigating CTSA websites to access CEnR resources, and CEnR competency nomenclature was not standardized. DISCUSSION/SIGNIFICANCE OF IMPACT: Our findings guide CEnR stakeholders to identify CEnR resources accessible online and gaps to address in CEnR resource development. Standardized nomenclature for CEnR competencies is needed for effective CEnR resource classification. Uniform organization of CTSA websites may maximize navigability. CONFLICT OF INTEREST DESCRIPTION: In addition to the funding information listed previously (see above), within the last three years, R.J. Piasecki has been employed as: Project Coordinator, CEnR Online Learning Project, Johns Hopkins University School of Nursing (Current) Temporary Employee (Doctoral Student Intern), Michigan State University Institute for Health Policy (Current) Clinical RN, Intrastaff at the Johns Hopkins Health System (Past) Research Data Analysis Assistant, Maryland Institute for Emergency Medical Services (Past - contracted)

